# Integrating Physiology, Transcriptome, and Metabolomics Reveals the Potential Mechanism of Nitric Oxide Concentration-Dependent Regulation of Embryo Germination in *Sorbus pohuashanensis*

**DOI:** 10.3390/plants14030344

**Published:** 2025-01-23

**Authors:** Caihong Zhao, Yue Zhang, Ling Yang

**Affiliations:** 1 State Key Laboratory of Tree Genetics and Breeding, Northeast Forestry University, Harbin 150040, China; zhaocaihong@nefu.edu.cn (C.Z.); zhangy-dl@nefu.edu.cn (Y.Z.); 2 College of Forestry, Beijing Forestry University, Beijing 100091, China

**Keywords:** nitric oxide, seed dormancy, *Sorbus pohuashanensis*, transcriptome, metabolome, hormones

## Abstract

Nitric oxide (NO) breaks a seed’s dormancy and stimulates germination by signaling. However, the key physiological metabolic pathways and molecular regulatory mechanisms are still unclear. Therefore, this study used physiological, transcriptomic, and metabolomics methods to analyze the key genes and metabolites involved in the NO regulation of plant embryo germination and their potential regulatory mechanisms. The physiological analysis results indicate that the appropriate concentration of NO increased the content of NO and hydrogen peroxide (H_2_O_2_) in cells, stimulated the synthesis of ethylene and jasmonic acid (JA), induced a decrease in abscisic acid (ABA) content, antagonistic to the gibberellin (GA_3_) effect, and promoted embryo germination and subsequent seedling growth. However, the high concentrations of NO caused excessive accumulation of H_2_O_2_, destroyed the reactive oxygen species (ROS) balance, and inhibited embryo germination and seedling growth. The combined analysis of transcriptomics and metabolomics showed that the genes related to phenylpropanoid (phenylalanine ammonia-lyase, trans-cinnamate 4-monooxygenase, ferulate-5-hydroxylase, coniferyl-alcohol glucosyltransferase), and flavonoid synthesis (10 genes such as *CHS*) were significantly up-regulated during embryo germination. The high concentration of exogenous NO inhibited embryo germination by up-regulating the expression of 4-coumaric acid coenzyme A ligase (4CL) and negatively regulating the expression of flavonoid synthesis genes. This suggests that NO concentration-dependently regulates phenylpropanoid and flavonoid biosynthesis, thereby affecting ROS metabolism and hormone levels, and ultimately regulates the dormancy and germination of *Sorbus pohuashanensis* embryos.

## 1. Introduction

*Sorbus pohuashanensis* has a high ornamental value [1]. Its seeds have a deep dormancy [2]. Sodium nitroprusside (SNP) can release NO, which is a potent plant regulatory substance that affects the content and metabolism of ROS and NO in cells under light conditions [3,4,5]. A large number of studies have reported that exogenous NO donor-SNP can relieve seed dormancy and promote germination by increasing NO levels [6,7,8]. However, higher concentrations of exogenous NO may affect the biosynthesis pathway of phenylpropanoids by inhibiting the expression and enzyme activity of some key genes, thereby inhibiting seed germination [9]. Therefore, the concentration of NO is one of the key factors affecting seed dormancy and germination.

NO regulates seed germination with the production of reactive oxygen species (ROS) and reactive nitrogen species (RNS), which is a necessary signal from dormancy to germination [10]. Many studies have confirmed that NO may have dual functions. For example, low concentrations of NO break the embryo dormancy of *S. pohuashanensis (Sorbus pohuashanensis)* by relying on ethylene synthesis [6]. This hormone-related signaling pathway is closely related to the accumulation of reactive oxygen species and antioxidant defense responses [11,12]. However, a high concentration of NO has a complex effect on seed germination, which can not only promote germination and enhance stress resistance but also may inhibit seed germination due to the high concentration. In addition, the promotion or inhibition of seed germination by NO is related to the role of ROS metabolism-related enzymes [13]. For instance, peroxisomes can produce NO and participate in lipid metabolism, amino acid oxidation, and ROS metabolism [14]. Catalase (CAT) can be activated by phosphorylation, which enhances its catalytic ability to scavenge ROS [15]. The physiological response of seed germination, which is regulated by NO, can be explained by the changes in the transcriptional abundance of enzymatic and non-enzymatic antioxidant defense genes [16,17]. Furthermore, the response and adaptation of plant embryos to NO is a very complex process involving the regulation of numerous genes in different metabolic pathways. NO and ROS can act independently in the same signaling pathway and have similar downstream responses and outcomes, but their internal molecular regulatory mechanisms need to be further investigated [18].

In this work, we used RNA sequencing (RNA-seq) and UPLC-MS/MS technology to provide an integrated map of transcriptomics and metabonomics of *S. pohuashanensis* embryos to fill the gaps in understanding the gene expression and metabolic regulation during the dormancy release and germination of *S. pohuashanensis* embryos caused by exogenous NO. We have provided a large body of evidence that the regulation of NO on *S. pohuashanensis* embryo germination is activated by a combination of gene transcription and metabolic homeostasis, particularly through the regulation of reactive nitrogen species (RNS), ROS, and hormones. These results help us to understand the mechanism of NO concentration-dependent regulation of embryo germination in *S. pohuashanensis*. It provides a reference for the study of dormancy release and plant seed germination at the multi-omics level.

## 2. Results

### 2.1. Phenotypic Concentration-Dependent Response to NO on Embryo Germination of S. pohuashanensis

**Figure 1:**
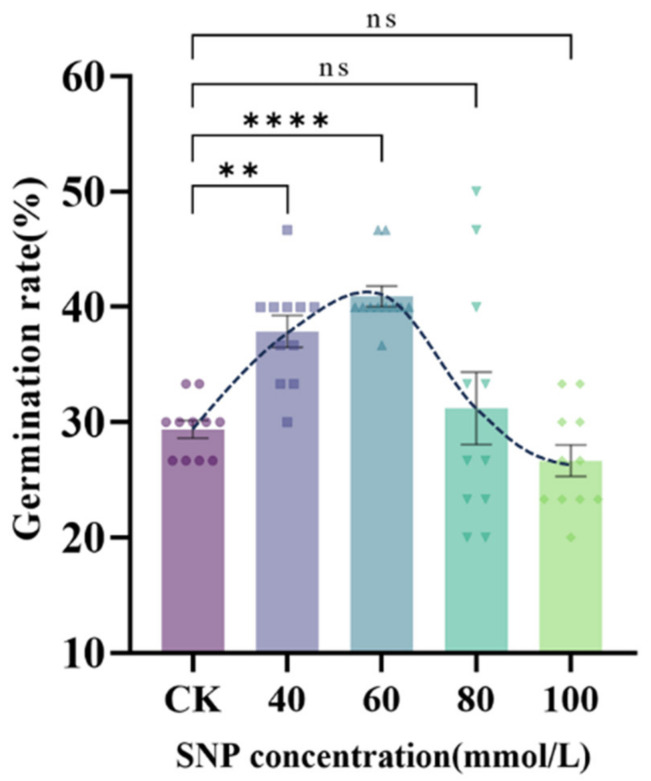
Effect of different concentrations of SNP on the embryo germination rate of *Sorbus pohuashanensis*. (Note: ns means *p* > 0.05, ** means *p* ≤ 0.01, **** means *p* ≤ 0.0001).

To help understand how SNP affects the growth of *S. pohuashanensis* embryos, we used a growth model in which *S. pohuashanensis* embryos were cultured in 5 mL of a sodium nitroprusside solution (0~100 mmol/L) for 8 days. We used a growth model to culture the embryos of *S. pohuashanensis* in 5 mL SNP if solution (0~100 mmol/L) for 8 days. The results showed that SNP had a biphasic concentration effect on the dormancy release of *S. pohuashanensis* seeds. Among them, compared with the control, the 40 mmol/L and 60 mmol/L SNP treatments significantly increased the embryo germination rate of *S. pohuashanensis* (increased by 28.9% and 39.2%, respectively), while 80 mmol/L and 100 mmol/L SNP treatments decreased by 6.2% and 9.3%, respectively (Figure 1).

**Figure 2:**
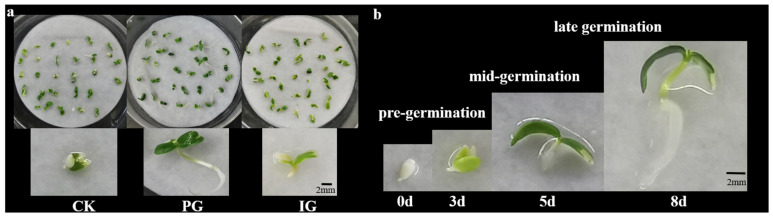
Germination model of an embryo of *Sorbus pohuashanensis*. (**a**) The phenotype of *Sorbus pohuashanensis* embryos cultured to day 8 after treatment with different SNP concentrations; (**b**) There are three stages of embryo germination of *Sorbus pohuashanensis*: 0–3 d is pre-germination, 3–5 d is mid-germination, and 5–8 d is late-germination.

At the same time, we also found that, compared with untreated (CK, water), the embryos of *S. pohuashanensis* treated with 60 mmol/L of SNP (PG for short) germinated faster, the seedlings grew healthily, and the leaves developed well. The embryos of *S. pohuashanensis* treated with 100 mmol/L of SNP (IG for short) germinated faster in the pre-germination stage, and the seedlings grew poorly and yellowed in the mid-germination stage (Figure 2a). In this study, the germination process was divided into three stages (Figure 2b): pre-germination (0–3 d), mid-germination (3–5 d), and late-germination (5–8 d). During the pre-germination stage, the two cotyledons began to stretch slightly and turned pale green. At the mid-germination stage, the color of the two cotyledons continues to turn green, the opening angle of the cotyledons increases, accompanied by the elongation of the hypocotyl, and the radicle begins to appear. At the late-germination stage, the hypocotyls and radicles elongated rapidly.

### 2.2. Physiological Response to NO in Embryo Germination of S. pohuashanensis

#### 2.2.1. The Response of NO-Dependent Concentration to RNS During the Embryonic Germination of *S. pohuashanensis*

**Figure 3:**
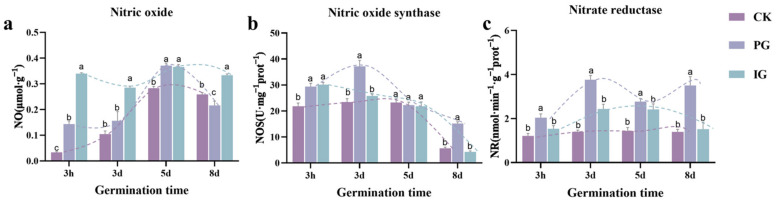
Effect of different concentrations of SNP treatment on the RNA content of *Sorbus pohuashanensis* embryos. (**a**) NO content. (**b**) NOS. (**c**) NR. Note: Different small letters in the figure indicate significant differences between the different treatments (*p* < 0.05).

To investigate the dynamic changes in physiological responses of *S. pohuashanensis* embryos during germination, NO content, nitric oxide synthase (NOS), and nitrate reductase activities (NR) were measured at 3 h, 3 d, 5 d, and 8 d after germination. The endogenous NO content increased continuously during the germination of the control embryos (Figure 3a, CK). At the pre-germination and mid-germination stages, the endogenous NO content of the embryos increased after treatment with exogenous NO (PG and IG) (compared to CK) but decreased at the late-germination stage (Figure 3a). At the pre-germination stage (3 h–3 d), PG treatment significantly increased endogenous NOS (Figure 3b) and NR (Figure 3c) activities (compared to CK), and thereafter NOS and NR decreased (but not less than the control). After 3 h of IG treatment, NOS activity was significantly higher than that of CK, and thereafter, there was no significant difference from CK (Figure 3b), while NR activity always showed no significant difference from CK (Figure 3c).

#### 2.2.2. The Response of NO-Dependent Concentration to ROS During the Embryonic Germination of *S. pohuashanensis*

**Figure 4:**
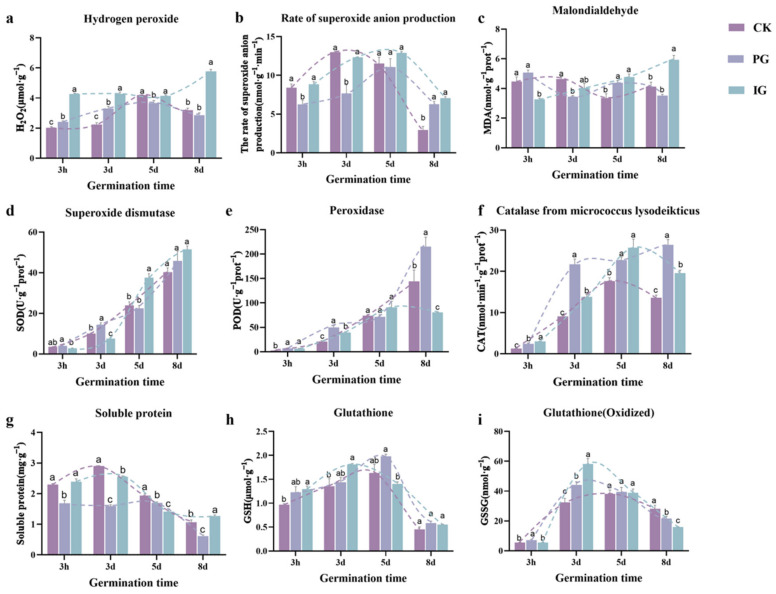
Effect of different SNP concentrations on the content of reactive oxygen species in the embryos of *Sorbus pohuashanensis*. (**a**) H_2_O_2_ content. (**b**) superoxide anion formation rate. (**c**) malondialdehyde content. (**d**) SOD content. (**e**) POD content. (**f**) CAT content. (**g**) soluble protein content. (**h**) glutathione (**i**) glutathione (oxidized). Note: Different small letters in the figure indicate significant differences between the different treatments (*p* < 0.05).

To investigate the dynamic changes of ROS during germination of the embryo of *S. pohuashanensis* under different concentrations of NO, the levels of H_2_O_2_ and superoxide anions, as well as the activities of the corresponding enzymes, were measured during germination. The content of H_2_O_2_ and superoxide anion in CK and PG first increased and then decreased, with PG and IG significantly increasing the content of H_2_O_2_ and decreasing the content of superoxide anion in the pre-germination stage (Figure 4a) and the content of H_2_O_2_ and superoxide anion in IG was significantly higher than that in CK in the late-germination stage.

During pre-germination, PG significantly increased the activities of enzymes related to reactive oxygen species, such as SOD, POD, and CAT. During the late-germination stage, the activities of POD and CAT were significantly higher in PG-treated embryos than those of CK and IG. In addition to affecting the activity of active oxygen-scavenging enzymes, NO treatment also increased the levels of antioxidant substances (GSH and GSSG) and showed a “ first increasing and then decreasing” trend in the germination process.

#### 2.2.3. The Concentration-Dependent Hormone Response to NO on Embryo Germination of *S. pohuashanensis*

**Figure 5:**
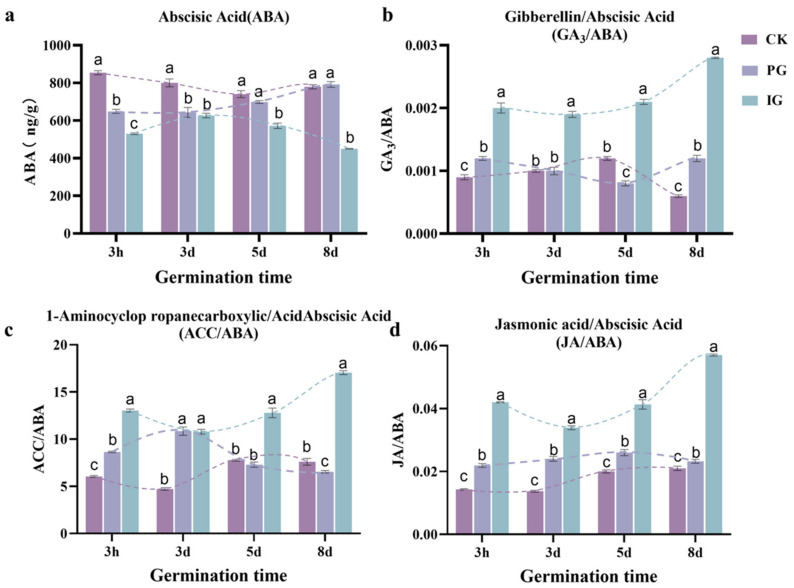
Effect of different concentrations of SNP treatment on the hormone content of *Sorbus pohuashanensis* embryos. (**a**) ABA content. (**b**) GA_3_/ABA. (**c**) ACC/ABA. (**d**) JA/ABA. Note: Different small letters in the figure indicate significant differences between the different treatments (*p* < 0.05).

The levels of ABA, GA_3_, ACC, and JA were determined to investigate the concentration-dependent regulation of the hormone balance during germination of the embryo *S. pohuashanensis* (Figure 5). The ABA content of embryos treated with PG and IG was higher than that of the control. GA_3_/ABA and JA/ABA of Ig treatment were significantly higher than those of CK and PG. With increasing germination time, JA/ABA of IG treatment first decreased and then increased, while CK and PG first increased and then decreased (consistent with the biphasic concentration–response curve).

**Figure 6:**
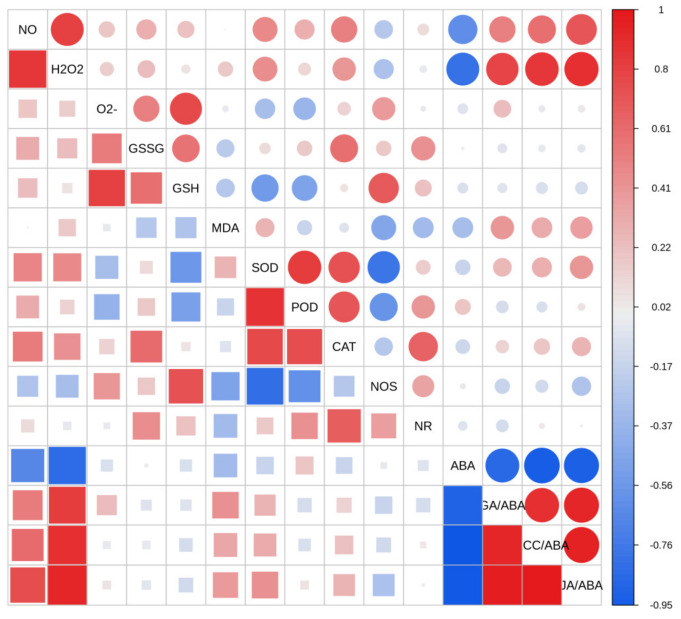
Correlation analysis of RNS, ROS, and hormones. Note, the diagonal represents the sample name. The color from blue to red indicates the change of correlation from negative to positive. The larger the figure of the upper and lower triangular regions, the greater the correlation, and vice versa.

The results of the correlation analysis showed (Figure 6) that the NO content of *S. pohuashanensis* embryos had a significant positive correlation with H_2_O_2_ and JA and a significant negative correlation with ABA. There was a significant positive correlation between NO content and H_2_O_2_ content in *S. pohuashanensis* embryos. H_2_O_2_ was positively correlated with JA, GA_3_, and ACC and negatively correlated with ABA.

### 2.3. Summary of Transcriptome Analysis

**Figure 7:**
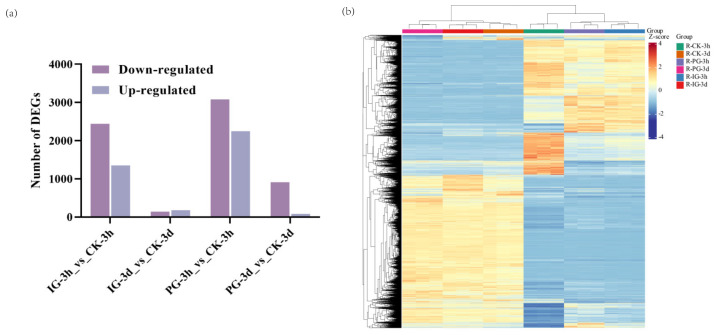
Effects of different concentrations of NO treatment on the transcriptome changes of *Sorbus pohuashanensis* embryos. (**a**) The number of differential genes in *Sorbus pohuashanensis*. (**b**) Differential enrichment analysis of *Sorbus pohuashanensis*.

In this study, we selected embryos treated with CK, PG, and IG at 3 h and 3 d in the pre-germination stage as experimental materials for transcriptome sequencing analysis. A total of 43,304 differentially expressed genes were identified by RNA-seq analysis (clean reads; q30 ≥ 91.76%; see Table 1 for a summary of RNA sequencing data). The GC contents of these sequences were higher than 45.96%, indicating that the sequencing results were of high quality. By pairwise comparison, the differentially expressed genes DEGs were determined between exogenous NO treatments with different concentrations (Figure 7a). Compared to CK-3h, PG-3h and IG-3h up-regulated 2247 and 1355 genes and down-regulated 3083 and 2440 genes, respectively. Compared to CK-3d, PG-3d and IG-3d up-regulated 85 and 183 genes and down-regulated 911 and 143 genes, respectively. Interestingly, the number of differentially expressed genes was significantly higher 3 h after embryo germination was significantly higher than after 3 days. All DEGs are divided into two categories, one grouped by the DEGs in 3 h and the other by the DEGs in 3 d (Figure 7b).

### 2.4. Differentially Expressed Genes Analysis

The results of KEGG and GO enrichment showed that the non-overlapping DEGs of NO concentration-dependently promoted the embryonic germination of *S. pohuashanensis* and were significantly enriched in lipid synthesis and metabolism, carbohydrate metabolism, amino acid biosynthesis, and metabolism, while the non-overlapping DEGs of NO-concentration-dependently inhibited the embryonic germination of *S. pohuashanensis* and were significantly enriched in plant hormone signal transduction, flavonoid biosynthesis, amino acid metabolism, and other pathways (Supplementary Material Figures S1 and S2). In PG-3d compared to CK-3d and IG-3d compared to CK-3d, the NO-dependent, non-overlapping DEGs that promoted embryo germination were significantly enriched in the MAPK signaling pathway, protein processing in the endoplasmic reticulum and plant hormone signaling, while non-overlapping DEGs that inhibited embryo germination were significantly enriched in fructose and mannose metabolism, pentose and glucuronate interconversions, plant hormone signaling transduction, and other pathways.

In summary, we found that exogenous NO can promote seed germination by regulating lipid metabolism, sugar conversion, and carbohydrate metabolism. The DEGs that inhibit germination regulate embryo germination mainly via plant hormone signaling, sugar conversion, and other metabolic pathways.

### 2.5. Validation of Differentially Expressed Genes by qRT-PCR

**Figure 8:**
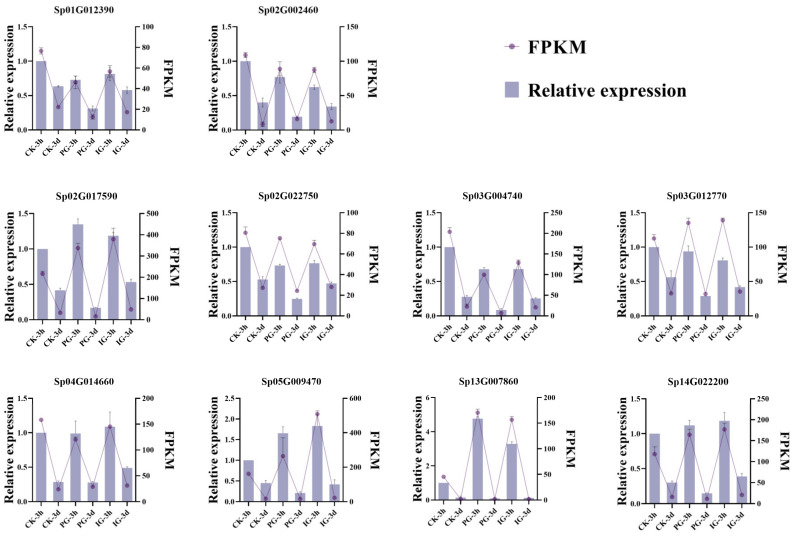
Verification of differential genes by qRT-PCR.

There was no significant difference between RNA-Seq and qRT-PCR data of *S. pohuashanensis*, and the trend was similar. Therefore, it was confirmed that the RNA-Seq data used in this study were reliable (Figure 8).

### 2.6. Metabolome Analysis

**Figure 9:**
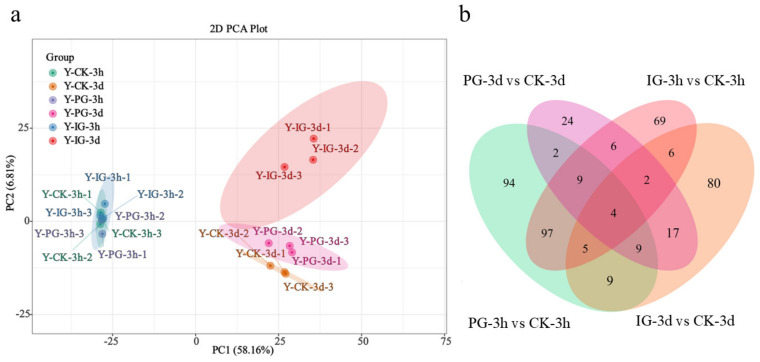
(**a**) PCA scores were detected in *Sorbus pohuashanensis* embryos at 3 h and 3 d after different concentrations of SNP treatment. PCA scores were derived from all metabolites detected in the three replicate samples under each treatment. (**b**) Venn diagram of DAMs among various pair-wise comparisons.

Using metabolomics analysis, the PCA method was used to compare the metabolite peaks detected between 3 h and 3 d after different concentrations of SNP treatment (Figure 9), and samples were separated into PC1 (58.16%) and PC2 (6.81%). The levels of metabolites in the samples of 3 h and 3 d were separated, indicating that there was a significant difference in metabolism between the samples of 3 h and 3 d. The metabolites of PG, IG, and CK in the 3 d samples were separated from each other, but the content of metabolites in the 3 h samples was summarized, indicating that the germination of seeds at the 3 d level changed significantly at the metabolic level. This shows that the transient effect of NO makes the change in transcriptome faster than the change in metabolite level.

**Figure 10:**
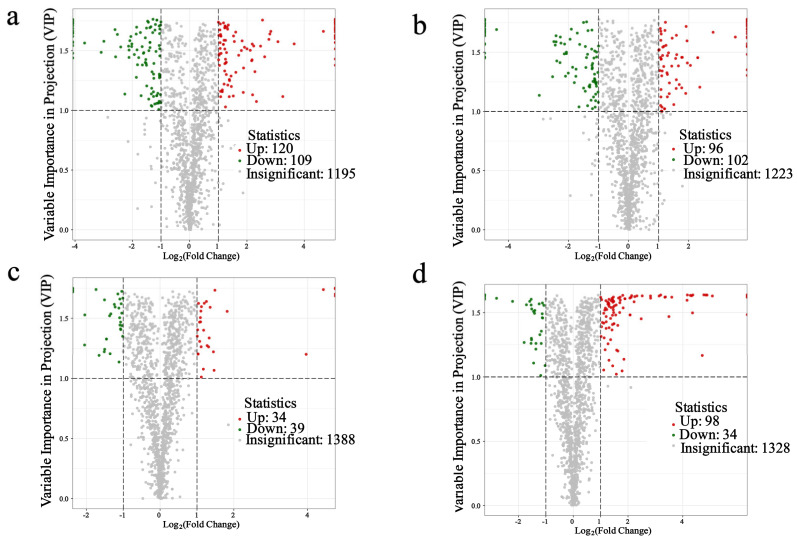
Difference analysis of gene expression after treatment with different concentrations of SNP. (**a**) PG-3h vs. CK-3h; (**b**) PG-3d vs. CK-3d; (**c**) IG-3h vs. CK-3h; (**d**) IG-3d vs. CK-3d.

Subsequently, the differentially expressed metabolites were identified by comparing three replicate samples treated with different SNP concentrations for 3 h and 3 d (Figure 10). A total of 229 metabolites (120 up-regulated and 109 down-regulated) were screened between PG-3h and CK-3h, 73 metabolites (34 up-regulated and 39 down-regulated) were screened between PG-3d and CK-3d, 198 metabolites (96 up-regulated and 102 down-regulated) were screened between IG-3h and CK-3h, and 132 metabolites (98 up-regulated and 34 down-regulated) between IG-3d and CK-3d.

To analyze the function of DAMs, the major differentially expressed metabolites of NO that concentration-dependently regulate the dormancy of *S. pohuashanensis* embryos for 3 h, we compared the differentially expressed metabolites of PG vs. CK and IG vs. CK (Supplementary Material Figure S3). In the non-overlapping-differential metabolites of PG-3h vs. CK-3h and IG-3h vs. CK-3h, the differential metabolites of NO concentration-dependently promoted embryo germination of *S. pohuashanensis* and were mainly enriched in lipid metabolism, biosynthesis of secondary metabolites and biosynthesis of amino acids. The differential metabolites of NO that concentration-dependently inhibited the embryo germination of *S. pohuashanensis* were mainly enriched in the biosynthesis of flavone and flavonol biosynthesis, amino acid metabolism, and carbohydrate pathways. It is hypothesized that low concentrations of exogenous NO may promote the germination of *S. pohuashanensis* embryos through the biosynthesis of amino acids. If the concentration of exogenous NO is too high, it may inhibit embryo germination through amino acid metabolism. This is consistent with the results of transcriptomics. In the non-overlapping differential metabolites of PG-3d vs. CK-3d and IG-3d vs. CK-3d, the differential metabolites of NO that concentration-dependently promoted the embryo germination of *S. pohuashanensis* were mainly enriched in the biosynthesis of secondary metabolites, while the differential metabolites of NO the concentration-dependently inhibited the embryo germination of *S. pohuashanensis* were mainly enriched in lipid metabolism, biosynthesis of secondary metabolites, amino acid metabolism, and other metabolic pathways. By analyzing different metabolites between 3 h and 3 d, the results showed that for embryos treated with NO for 3 h, DAMs were significantly enriched in phenylpropane and flavonoid biosynthesis, lipid metabolism, and phenylalanine metabolism (also in a concentration-dependent manner). The DAMs treated for 3 days were significantly enriched in flavonoid and flavonol biosynthesis, phenylalanine metabolism, and other metabolic pathways.

These results suggest that changes in metabolite accumulation are strictly controlled by differential gene expression. Flavonoid biosynthesis and phenylpropanoid biosynthesis are considered to be the major metabolic pathways of exogenous NO affecting embryo germination. Therefore, we analyzed the metabolites and crucial genes involved in NO-related metabolic pathways and investigated the mechanism of NO’s concentration-dependent effect on dormancy selection of dormancy and germination of *S. pohuashanensis* embryos.

### 2.7. Comprehensive Metabolome and Transcriptome Analysis of Differential Regulation of Key Genes and Metabolites

**Figure 11:**
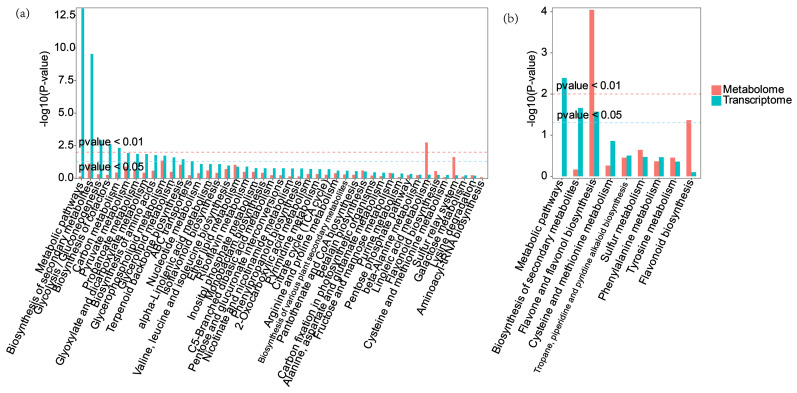
KEGG pathways of DEGS and DAMs. (**a**) PG-3h vs. CK-3h. (**b**) IG-3h vs. CK-3h.

**Figure 12:**
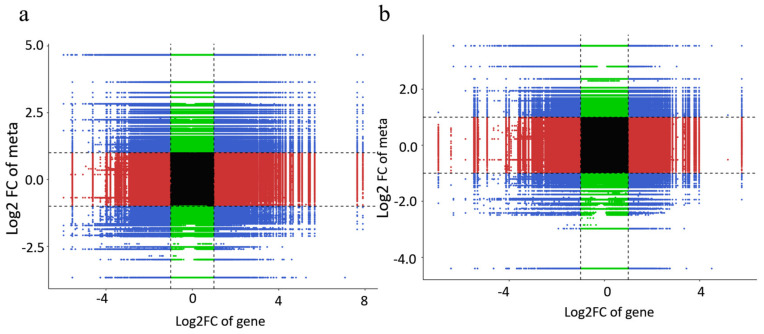
Transcriptome and metabolome combined analysis of nine quadrants. The black dotted line, from left to right, from top to bottom, is divided into 1-9 quadrants. (**a**) PG-3h vs. CK-3h, (**b**) IG-3h vs. CK-3h.

To further analyze the DEGs and DAMs, we annotated them in the KEGG database and found many joint common pathways (Figure 11). PG-3h vs. CK-3h was enriched in 45 pathways, while IG-3h vs. CK-3h was enriched in 9 pathways. Among these pathways, the flavonoid biosynthetic pathway and the phenylpropanoid biosynthetic pathway were highly enriched pathways under PG and IG treatments. The correlation coefficients of the nine quadrants showed that the differential expression of genes and metabolites was consistent in the third and seventh quadrants (Figure 12). We conclude that these genes are positively correlated with the regulation of metabolites and that the changes in metabolites may be positively regulated by these genes.

### 2.8. The Role of Differential Genes and Differential Metabolites in Phenylpropanoid Biosynthesis and Flavonoid Biosynthesis Pathways

Considering all the results of the transcriptome and metabolome, we focused on the genes and metabolites that showed significant differences between PG-3h and IG-3h. The results showed that phenylpropanoid and flavonoid genes/metabolites changed significantly after treatment with different concentrations of exogenous NO. We hypothesize that these genes and metabolites may play a different role in the response of PG and IG to pre-embryonic germination.

**Figure 13:**
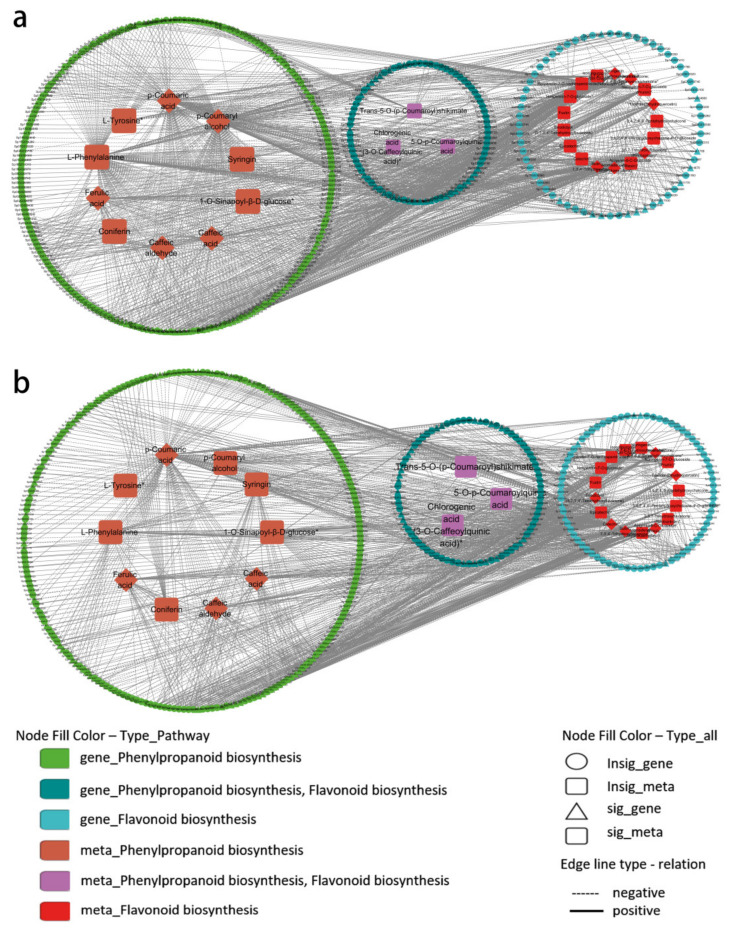
Regulatory co-expression network of different genes and different metabolites related to the biosynthesis of phenylpropanoids and flavonoids. The Cytoscape software (version 3.10.0) was used to visualize the network. (**a**) PG-3h vs. CK-3h, (**b**) IG-3h vs. CK-3h.

Therefore, we performed a Pearson correlation analysis on 450 genes and 30 metabolites in the phenylpropanoid and flavonoid biosynthetic pathways and constructed a correlation network diagram of the different genes and the different metabolites. Based on |r| > 0.8, *p* < 0.05 as the threshold, the regulatory co-expression network was visualized in Cytoscape (Figure 13). The results show that a total of 390 nodes were connected to 1365 edges of PG-3h, and 405 nodes were connected to 1109 edges of IG-3h. In the comparison between PG-3h and CK-3h, there were 62 key gene nodes: 5 key metabolite nodes significantly associated with phenylpropanoid biosynthesis, 11 key gene nodes, 6 key metabolite nodes associated with flavonoid biosynthesis, and 11 key gene nodes involved in both phenylpropanoid biosynthesis and flavonoid biosynthesis. In IG-3h compared to CK-3h, there were 47 key gene nodes: 4 key metabolite nodes related to the phenylpropanoid biosynthetic pathway, 21 key gene nodes, 6 key metabolite nodes involved in flavonoid biosynthesis pathway, and 6 key gene nodes related to both the phenylpropanoid and flavonoid biosynthetic pathway. We hypothesize that these genes and metabolites may play a key role in the response of exogenous NO’s concentration-dependent effects on the release of dormancy in *S. pohuashanensis* embryos.

Based on the expression patterns of key genes and metabolites, we constructed models for the phenylpropanoid and flavonoid biosynthetic pathways. About 93 key DEGs and 5 DAMs were involved in the phenylpropanoid biosynthetic pathway, and about 37 key DEGs and 6 DAMs were involved in the flavonoid biosynthetic pathway. About 68 major DEGs and 4 DAMs were involved in the phenylpropanoid biosynthetic pathway, and about 27 major DEGs and 6 DAMs were involved in the flavonoid biosynthetic pathway.

**Figure 14:**
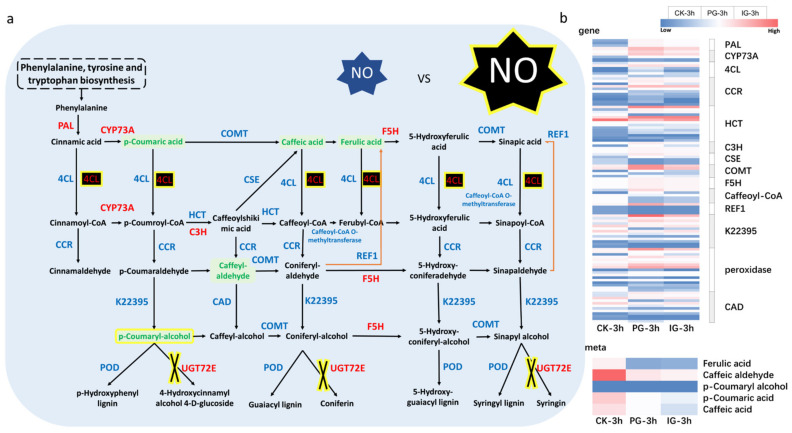
Phenylpropanoid biosynthetic pathway. (**a**) NO concentration-dependent regulation of phenylpropanoid biosynthesis signaling pathway during embryo germination of *Sorbus pohuashanensis*. The blue frame indicates PG-3h vs. CK-3h, and the yellow frame indicates that IG-3h vs. CK-3h influences the pathway of phenylpropanoid biosynthesis compared to PG-3h vs. CK-3h. Red indicates upregulated genes/metabolites, blue indicates upregulated and downregulated genes/metabolites, and green indicates downregulated genes/metabolites. (**b**) Heatmap of gene and metabolite expression levels. Note, PAL, phenylalanine ammonia-lyase; CYP73A, trans-cinnamate 4-monooxygenase; 4CL, 4-coumarate-CoA ligase; CCR, cinnamoyl-CoA reductase; HCT, shikimate O-hydroxycinnamoyltransferase; CSE, caffeoylshikimate esterase; COMT, caffeic acid 3-O-methyltransferase/acetylserotonin O-methyltransferase; CYP98A(C3′H), 5-O-(4-coumaroyl)-D-quinate 3′-monooxygenase; K22395, cinnamyl-alcohol dehydrogenase; POD, peroxidase; UGT72E, coniferyl-alcohol glucosyltransferase; CAD, cinnamyl-alcohol dehydrogenase; REF1, coniferyl-aldehyde dehydrogenase; CYP84A (F5H), ferulat-5-hydroxylase.

Under exogenous NO treatment, a large number of phenylpropanoid biosynthesis genes were upregulated, and their expression levels were significantly higher than those of the control (Figure 14). Compared with CK treatment, PG significantly promoted phenylalanine ammonia-lyase [EC: 4.3.1.24] and trans-cinnamate 4-monooxygenase [EC: 1.14.14.91] in the phenylpropanoid biosynthetic pathway. The expression of ferulate5-hydroxylase [EC: 1.14.-.-] and coniferyl-alcohol glucosyltransferase [EC: 2.4.1.111] was significantly inhibited by IG treatment, while the expression of 4-coumarate-CoA ligase [EC: 6.2.1.12] and coniferyl-alcohol glucosyltransferase [EC: 2.4.1.111] was significantly inhibited by IG treatment. The key metabolites, such as p-coumaric acid, caffeic acid, ferulic acid, and caffeoyl-aldehyde, were down-regulated overall. Therefore, different concentrations of exogenous NO treatment may continue to affect the metabolism of phenylpropanoids. The higher the concentration of exogenous NO, the lower the expression of the metabolites.

**Figure 15:**
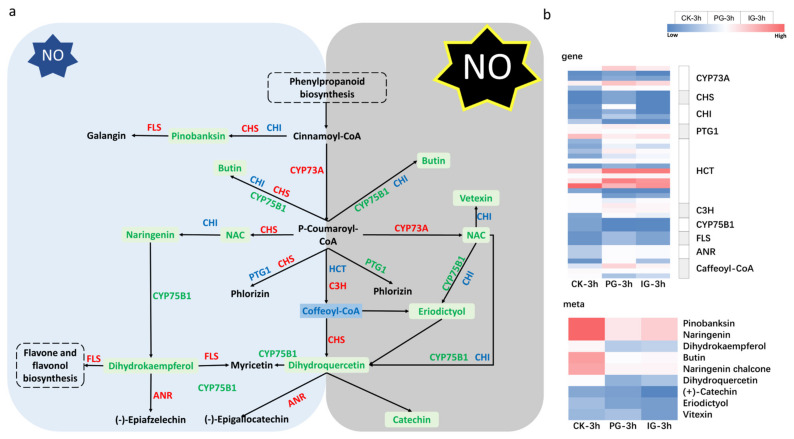
The pathway of flavonoid biosynthesis pathway. (**a**) NO concentration-dependent regulation of flavonoid biosynthesis signaling pathway during embryo germination of *Sorbus pohuashanensis*. The blue outer frame indicates PG-3h compared to CK-3h, and the grey outer frame indicates IG-3h compared to CK-3h. (**b**) Heatmap of gene and metabolite expression levels. Note, CHI, chalcone isomerase; CHS, chalcone synthase, FLS, flavonol synthase; NAC, naringenin chalcone; CYP75B1, flavonoid 3′-monooxygenase; CYP73A, trans-cinnamate 4-monooxygenase; HCT, shikimate O-hydroxycinnamoyl transferase; CYP98A(C3′H), 5-O-(4-coumaroyl)-D-quinate 3′-monooxygenase; ANR, anthocyanidin reductase; PTG1, phlorizin synthase.

Compared to CK-treated embryos, most of the genes involved in flavonoid biosynthesis were upregulated by exogenous NO (Figure 15). With the increase in NO concentration, the expression levels of key genes such as trans-cinnamate 4-monooxygenase, 5-O-(4-coumaroyl)-D-quinate 3‘-monooxygenase, flavonol synthase, and anthocyanidin reductase were decreased. This shows that exogenous NO upregulates the up-regulation of flavonoid synthesis genes, but the higher the concentration, the lower the series of flavonoid synthesis genes.

## 3. Discussion

### 3.1. NO Concentration-Dependently Regulates ROS Signaling During Embryo Dormancy Release in S. pohuashanensis

NO is regulated by both environmental and endogenous signaling and has both synergistic and antagonistic effects. It is a free radical that can effectively regulate physiological responses in plant growth and development [19,20,21,22]. Our results showed that PG could promote the embryo germination of *S. pohuashanensis*, but embryo germination and seedling growth and development were inhibited after IG treatment. This biphasic concentration effect is consistent with previous reports on seed germination in *Arabidopsis thaliana* (L.) Heynh. [4,23], apple (*Malus pumila* Mill.) [24], barley (*Hordeum vulgare* L.) [4], *Paulownia elongata* S. Y. Hu [25] and many halophytes [26]. At the same time, the NO content in *S. pohuashanensis* embryos increased after SNP treatment for 3 h, which may be due to the increase in endogenous NO level in embryos by exogenous NO through oxidation or reduction pathway [27] and the increase in NO content may come from enzyme or non-enzyme pathway [28]. The results of Krasuska showed that the transition of apple flowers from dormancy to germination is related to the increase in NOS activity [10]. This is in line with our results. Due to the high activity and instability of NO, the existence of NOS in the context of its synthesis in higher plants is very elusive. Moreover, it is not yet clear at the molecular level how NO concentration regulates embryonic dormancy release and germination [10]. In the stage of pre-germination, in this study, the decrease in nitrate reductase activity caused by high concentrations of nitric oxide may be due to the participation of NR in the synthesis of NO. Exogenous NO controls its production by regulating GSNOR activity and nitrate assimilation to reduce NR activity [19,29,30].

ROS (such as H_2_O_2_, superoxide anions, etc.) and NO play a crucial role in regulating the dormancy and germination of plant seeds [31]. Low levels of ROS act as signaling molecules that promote the abrogation of dormancy and trigger seed germination [8]. Excessive accumulation of ROS accumulation can lead to the accumulation of reactive oxygen species in germinated seeds, and the change in the redox state of cells leads to the induction of gene expression [5]. In our results, PG treatment led to a significant increase in hydrogen peroxide content in embryos during embryo dormancy release, which positively regulated embryo germination and seedling morphogenesis. With increasing culture duration, treatment with a high concentration of exogenous NO led to a significantly higher H_2_O_2_ content than in the control and PG treatment. In the late stage of germination (8 d), the excessive accumulation of H_2_O_2_ in IG-treated *S. pohuashanensis* embryos destroyed the balance of ROS and affected the growth and development of seedlings in the late stage of seed germination. The ROS content depends not only on the ROS production system but also on the efficiency of the redox feedback mechanism between NO and ROS content [31], such as SOD. Many studies have shown that the activity of antioxidant enzymes increases during seed impregnation [32]. However, in most cases, the activation of the antioxidant system is a late event in the germination process, and the activity of antioxidant enzymes increases in the presence of stress [15]. In our results, the significant increase in SOD, POD, and CAT levels caused by treatment with a high concentration of exogenous NO on day 5 of embryo germination confirmed this point, and the significant changes in POD and CAT activities at the early and late stages of germination depended on the concentration of exogenous NO [14]. It is characterized by the active metabolism of ROS and is a target for NO-mediated PTM [33]. Treatment with PG significantly increased the CAT activity of *S. pohuashanensis* embryos during germination. Compared with PG, the significant inhibition of CAT by IG might mean that the ability of embryos to remove internal H_2_O_2_ is lower, which is closely related to the physiological or adverse processes leading to an increase in oxidative metabolism [14,34]. The effect was significant during embryo dormancy (on the third day of germination). The effect was significant during the dormancy release period of the embryo (the third day of germination). This result is consistent with our previous research results, findings suggesting that NO acts upstream of H_2_O_2_, thereby regulating CAT activity [35]. Similar studies in plants have also shown that NO donors can react with superoxide anions and inhibit enzyme activity [14,36].

Studies have shown that the process of cell division depends on the increase in glutathione levels during the transition from germination to seedling growth [37,38,39]. This is consistent with our findings that the appropriate concentration of SNP treatment resulted in an increase in GSH levels during the abrogation of embryonic dormancy. However, the excessive accumulation of GSSG is toxic, and the high concentration of exogenous NO treatment leads to the inhibition of embryonic germination and seedling growth of *S. pohuashanensis*.

### 3.2. NO Concentration-Dependently Regulates Hormone Levels During Embryo Dormancy and Germination in S. pohuashanensis

Plant hormones act together with the gas signaling molecules NO and ROS as second messengers to regulate seed germination and seedling growth after germination [5]. Seed germination and dormancy, although mainly dependent on hormone balance, are primarily regulated by abscisic acid, gibberellin, ethylene, and JA by maintaining appropriate ROS/RNS levels [40]. GA_3_ and ABA are considered to be the most important hormones counteracting the regulation of seed germination [41]. Similar to previous studies, our results also showed that NO concentration regulated ABA content in the embryo throughout seed germination and seedling growth and development. NO significantly promoted embryo germination and seedling growth by causing a decrease in ABA content, which counteracted the effect of GA_3_. Furthermore, the ABA content of the IG treatment was significantly lower than that of the control, while the GA_3_ content was significantly higher than that of the control. Our previous study found that NO significantly abolished embryo dormancy in *S. pohuashanensis*, which was related to the increase in ethylene synthesis [35]. In this experiment, the concentration-dependent effect of NO on endogenous ethylene content was studied. The results showed that IG could significantly promote the increase in ethylene content in the embryo of *S. pohuashanensis*, which was significantly higher than that of PG treatment. At the same time, in the period of embryonic dormancy, release, seedling growth, and development were significantly higher than that of the control. In this study, it was found that treatment with exogenous NO throughout germination and seedling morphogenesis exogenous NO treatment significantly increased the endogenous JA content of *S. pohuashanensis* embryos, and the JA content increased with the increase in exogenous NO concentrations. This indicates that NO concentration-dependently stimulated the biosynthesis of JA, which was an important step in the abrogation of embryonic dormancy and late seedling growth and development of *S. pohuashanensis* by NO. It is well known that JA can regulate multiple adaptive responses of plants to various environmental stresses [42]. In this study, the increase in JA content caused by the high concentration of exogenous NO may be due to the stress response of plants to excessive exogenous NO. However, the JA-mediated protective mechanism of the embryo caused by exogenous NO needs to be further investigated. In addition, in agriculture, plant hormones can regulate the genes encoding NR, NIR, GS, and GOGAT and the activities of corresponding enzymes in the process of NO regulating the absorption and transport of nitrogen by plants, thereby regulating nitrogen assimilation [43].

### 3.3. NO Concentration-Dependently Regulated the Changes of Key Genes and Metabolites in the Biosynthesis Pathway of Phenylpropanoid and Flavonoid in the Early Stage of Embryo Germination of S. pohuashanensis

We performed a comprehensive analysis of transcriptomes and metabolomes to determine the metabolites and metabolic pathways significantly affected by exogenous NO concentration-dependent regulation at the early stage of *S. pohuashanensis* embryo germination, especially phenylpropane and flavonoid biosynthesis related to seed germination [44]. Phenylpropanoids make an important contribution to NO regulation of *S. pohuashanensis* embryo germination. The flavonoids produced by phenylpropanoids have a strong antioxidant capacity and can scavenge ROS [45], protect seeds from oxidative damage, and play an important role in seed germination. Similarly, in our results, the differentially expressed genes in NO concentration-dependently promoted/inhibited embryo germination of *S. pohuashanensis* and were significantly enriched in the phenylpropanoid biosynthetic pathway. By comparing the nonoverlap of differential genes and differential metabolites in PG-3h vs. CK-3h and IG-3h vs. CK-3h, the specific metabolic pathways of NO’s concentration-dependent regulation of *S. pohuashanensis* embryos were determined. Our results showed that the differential genes of NO concentration-dependently promoted Sorbus embryo germination and were significantly enriched in the phenylpropanoid and flavonoid biosynthetic pathways. The differential genes of NO that concentration-dependently inhibited embryo germination of *S. pohuashanensis* were significantly enriched in phenylalanine metabolism and flavonoid and flavonol biosynthesis. The phenylpropanoid biosynthetic pathway begins with phenylalanine metabolism, so a high concentration of exogenous NO in embryo treatment can inhibit the upstream pathway of the phenylpropanoid biosynthetic pathway, thereby inhibiting embryo germination.

The genes related to phenylpropanoid biosynthesis (PAL, CYP73A, F5H, UGT72E) were significantly upregulated by an appropriate concentration of exogenous NO (PG) during the regulation of embryo germination of *S. pohuashanensis*. PAL is the first step of the phenylpropanoid pathway involved in the synthesis of phenolic compounds, such as phenolic acids, lignin, flavonoids, etc. It plays an important role in the formation of the cell wall and the connection between cells in the process of exogenous NO regulation of germination of *S. pohuashanensis* embryos [46]. UGT72E is mainly involved in the glycosylation process of phenylpropanoid compounds. In this study, UGT72E was significantly upregulated under PG treatment, but there was no significant difference under IG treatment, suggesting that an appropriate concentration of exogenous NO may affect the structure and function of the cell wall by regulating the glycosylation process and promote the embryo germination of *S. pohuashanensis* embryo. 4CL (4-coumaric acid-CoA ligase) is an important gene in phenylpropanoid biosynthesis, which affects the biosynthesis of flavonoids and plays an important role in plant physiology and plant protection [46]. In this study, we found that the expression of 4CL was significantly upregulated during embryonic germination of *S. pohuashanensis*, which is regulated by a high concentration of NO, in contrast to an appropriate concentration of exogenous NO, and that a high concentration of exogenous NO can inhibit embryonic germination by impairing the high expression of 4CL. In conjunction with our previous studies, we found that exogenous NO could significantly increase the ROS content in *S. pohuashanensis* embryos, suggesting that the reduction of p-coumaric acid, caffeic acid, ferulic acid, and caffeyl-aldehyde can stimulate the accumulation of ROS in *S. pohuashanensis* embryos, thereby inhibiting the germination of *S. pohuashanensis* embryos treated with high concentrations of exogenous NO.

Flavonoids are widely distributed secondary metabolites. In this study, embryo germination was influenced by the concentration of key genes and metabolites involved in the flavonoid biosynthetic pathway. CHS is the first reaction step responsible for catalyzing the flavonoid pathway. It is significantly upregulated in NO, dependent on concentration, to promote *S. pohuashanensis* embryo germination, but the difference is not significant under IG treatment, suggesting that the appropriate concentration of exogenous NO promotes the accumulation of the CHS gene family to promote seed germination. Similar to our results, the abrogation of seed dormancy of *Polygonatum sibiricum* significantly promoted the germination of the flavonoid biosynthetic pathway [47]. Most of the flavonoid biosynthetic genes were upregulated in the embryos of *S. pohuashanensis* treated with exogenous NO (Figure 13). However, with increasing NO concentration, the expression of key genes such as CYP73A, C3H, FLS, and ANR decreased. It is suggested that although exogenous NO causes upregulation of flavonoid synthesis genes, the higher the concentration, the lower the expression of a number of flavonoid synthesis genes decreases as the concentration increases. Flavonoids and phenylpropanoids play an important role in the elimination of ROS [48,49]. In this study, the appropriate concentration of exogenous NO treatment could promote germination by upregulating the accumulation of flavonoids and regulating the ROS content, while the higher concentration of exogenous NO negatively regulates the synthesis of flavonoid synthesis genes, thereby inhibiting embryo germination. Interestingly, our analysis of the metabolic results showed that metabolites associated with this pathway were significantly downregulated.

## 4. Materials and Methods

### 4.1. Plant Material

In October 2018, ripe berries were collected from adult mother trees of *S. pohuashanensis* in Maoershan Experimental Forest Farm (127°30′–127°34′ E, 45°21′–45°25′ N) in Shangzhi City, Heilongjiang Province, and the seeds were processed by water selection [50]. The plump, pure, and ripe seeds with a harmless moisture content (9–10%) were put into plastic bags and stored at 0–5 °C. The weight of 1000 seeds, water content, and viability were measured before the experiment and then imbibed in distilled water at 20 °C ± 5 °C for 48 h. After imbibition, the seeds were stirred and soaked in 0.2% (*v*/*v*) NaCl solution for 10 min, washed with clean water, and the seed coat was peeled off on ice (*S. pohuashanensis* is an endosperm-free seed) to obtain the naked embryo out for the experiment [6].

### 4.2. SNP Pretreatment and Embryo Germination Conditions

A Petri dish with a diameter of 35 mm was placed in the large Petri dish, and a filter paper completely soaked with 5 mL of 0.05 mol·L^−1^ Hepes-KOH (pH 7.0) was placed on top; 30 stripped embryos were placed on it. Then, the Petri dish with 5 mL of SNP solution (0, 40, 60, 80, or 100 mmol/L) was added to the same Petri dish, and, finally, the large Petri dish was sealed with a preservation film, and each treatment was repeated three times. Gaseous NO is released directly from the donor dish and acts on the embryos in the recipient dish. The embryos were irradiated with 25 °C and 60 μ mol·m^−2^·S^−1^ light for 3 h and germinated at16 h of light/8 h of darkness. The germination rate was calculated to determine the optimal SNP concentration after 8 days of germination in the culture room for 8 days. The standard for germination standard is that the cotyledons turn green, and the hypocotyl radicle elongation reaches 2 mm [5]. The calculation method of germination rate was based on the percentage of seed germination to the total number of tested seeds [6].

### 4.3. Determination of Related Indexes of Active Nitrogen and Active Oxygen

Tissue samples were taken 3 h and 1–8 days after SNP treatment. The relevant indicators of RNS are based on the method of Wang (2023) [6]. A total of 0.1 g fresh samples of embryos or seedlings were extracted and analyzed for RNS and ROS content.

### 4.4. Determination of Hormone Content (ABA, GA_3_, ACC, and JA)

Hormone levels (ABA, GA_3_, ACC, and JA) were detected by enzyme-linked immunosorbent assay (ELISA) with double antibody and one-step sandwich method. The absorbance (OD value) was determined at a wavelength of 450 nm, and the sample concentration was calculated [6,51].

### 4.5. Transcriptome Analysis

Total RNA was extracted from *S. pohuashanensis* embryos by ethanol precipitation and the CTAB-PBIOZOL method. After the library construction and detection were qualified, the DNB (DNA Nano Ball) was prepared and then loaded onto the sequencing chip to be sequenced with the high-throughput sequencer. The offline data was filtered to obtain clean data [52], which was sequenced with the reference genome of *S. pohuashanensis*.

### 4.6. Metabolomic Analysis

Using vacuum freeze-drying technology, the embryos of *S. pohuashanensis* were placed in a freeze-drying machine (Scientz-100 F, Scientz Group, Ningbo, China), and the samples were crushed into powder by (MM 400, Retsch, Haan, Germany) type pulverizer. Then, 50 mg of the sample powder was weighed using an electronic balance (MS105DΜ), and 1200 μL of −20 °C pre-cooled 70% methanolic aqueous internal standard extract was added (less than 50 mg at a rate of 1200 μL extractant per 50 mg sample). It was shaken once every 30 min for 30 s, for a total of 6 times. The treated samples were centrifuged and filtered for UPLC-MS/MS analysis.

The subsequent data processing was implemented by the statistical functions prcomp, cor, ComplexHeatmap 2.8.0, and MetaboAnalystR 1.0.1 in the software package R 3.5.1 (www.r-project.org).

### 4.7. Quantitative Real-Time PCR

To further verify the reliability of the differential expression results of each treatment group in *S. pohuashanensis* samples, we randomly selected 10 genes for qRT-PCR to verify gene expression changes. Using the actin gene of *S. pohuashanensis* as an internal reference, 13 target gene (Table 2 for primer information) primers were designed using Primer 6 software, and the relative expression was expressed by 2^−ΔΔCt^.

### 4.8. Statistical Analyses

Data from three biological replicates were analyzed using GraphPad Prism 9, Excel 2019, and SPSS 20.0 and are expressed as mean ± SD. Using one-way ANOVA, *p* < 0.05 was considered significant.

## 5. Conclusions

This study showed that exogenous NO concentrations affect embryo dormancy and germination of *S. pohuashanensis* in a biphasic concentration-dependent effect. In seed germination and seedling morphogenesis, appropriate NO concentrations promoted seed germination by increasing NO and H_2_O_2_ content, reducing ABA content, and increasing ethylene and JA content in *S. pohuashanensis* embryos. However, high concentrations of NO can increase the content of NO in *S. pohuashanensis* embryos. However, high concentrations of NO inhibited the activity of antioxidant enzymes in the embryo of *S. pohuashanensis*, disrupted the hormone balance of the embryo of *S. pohuashanensis*, and inhibited seed germination. A comprehensive multi-omics analysis revealed that NO concentration-dependently regulated *S. pohuashanensis* embryo germination, which was mainly regulated by biological pathways such as phenylpropanoid and flavonoid biosynthesis. Moreover, the biological events that occurred at the transcriptional level were consistent with the phenotype, hormone characteristics, and qRT-PCR analysis. This study provides the major target genes and metabolites of NO in regulating embryo germination of *S. pohuashanensis* and provides an important basis for the molecular mechanism of NO in seed germination of other Rosaceae plants.

## Figures and Tables

**Table 1:** The RNA-seq information of samples.

Sample	Raw Reads	Clean Reads	Q20 (%)	Q30 (%)	GC Content (%)
CK-3h-1	56,725,510	55,160,662	97.82	93.14	46.34
CK-3h-2	54,750,690	53,237,138	97.89	93.37	46.41
CK-3h-3	53,956,558	52,231,748	97.9	93.39	46.29
CK-3d-1	64,708,806	62,885,714	97.9	93.37	46.8
CK-3d-2	70,509,068	68,250,268	97.84	93.18	46.83
CK-3d-3	62,635,840	60,728,020	98	93.72	46.79
PG-3h-1	58,613,942	56,862,708	97.8	93.06	46.1
PG-3h-2	80,612,590	78,953,442	98.48	95.86	45.96
PG-3h-3	62,406,360	60,066,066	97.92	93.44	46.08
PG-3d-1	51,615,176	50,409,014	97.35	91.76	46.67
PG-3d-2	55,948,152	54,195,682	97.99	93.67	46.77
PG-3d-3	58,913,916	57,146,268	97.87	93.3	46.8
IG-3h-1	63,467,176	61,660,990	97.93	93.47	46.07
IG-3h-2	65,084,926	63,275,686	97.79	93.06	46.15
IG-3h-3	75,233,136	72,775,194	97.82	93.09	46.23
IG-3d-1	67,369,076	65,026,244	97.83	93.15	46.79
IG-3d-2	58,658,806	56,981,024	97.79	93.05	46.73
IG-3d-3	61,788,888	60,412,016	98.01	93.71	46.81

**Table 2:** qRT-PCR primers for candidate genes.

Gene_id	Forward Primers	Reverse Primers
Spβ-actin	TGGATGGCTGGAAGAGGA	GAGCGGGAAATTGTGAGG
Sp01G012390	GGAAAGCAGCCGAAATCT	TCTCCCACACACCGAAGT
Sp02G002460	GATGTGTATTGCCCTCCT	TCTCTTTACCACCGTTGA
Sp02G017590	GGGTCGGGTTACAAGCG	GGGCAAGAAAACTCGGTG
Sp02G022750	AGGGTCCTCCCCCATACA	CGCTCTCCTTAGCAGTCA
Sp03G004740	CAACCATCCCAAGCCACA	AAACTCCTCCCCTCCTCC
Sp03G012770	CCGTTTATTCCGCCATCA	GGCGTCCCTTTCTTCTCTT
Sp04G014660	GCACGGAGAGGCTAGAGA	GTTGCAGCGAACAATGGT
Sp05G009470	CTGTTCCATTCCTATCCC	CAAAACCCAGTACCCATC
Sp13G007860	TTCTCCGTGTTCGTCCAA	ACCGCCTCCTGTCATTCC
Sp14G022200	GCGGCGAACAAATAGGGT	TGGCGGATAAGGAACGGA

## Data Availability

The datasets supporting the conclusions of this article are included within the article and its Supplementary Files. All RNA-seq reads were deposited at NCBI (PRJNA1153935).

## Supplementary Materials

The following supporting information can be downloaded at: https://www.mdpi.com/article/10.3390/plants14030344/s1, Figure S1. KEGG analysis of DEGs during NO-dependent concentration regulation of embryo germination of Sorbus pohuashanensis. (a) KEGG enrichment statistics of PG-3h vs. CK-3h in non-overlapping DEGs of PG-3h vs. CK-3h and IG-3h vs. CK-3h. (b) KEGG enrichment statistics of IG-3h vs. CK-3h in non-overlapping DEGs of PG-3h vs. CK-3h and IG-3h vs. CK-3h. (c) KEGG enrichment statistics of PG-3h vs. CK-3h in non-overlapping DEGs of PG-3h vs. CK-3h and PG-3d vs. CK-3d. (d) KEGG enrichment statistics of PG-3d vs. CK-3d in non-overlapping DEGs of PG-3h vs. CK-3h and PG-3d vs. CK-3d. (e) KEGG enrichment statistics of IG-3h vs. CK-3h in IG-3h vs. CK-3h and IG-3d vs. CK-3d non-overlapping DEGs. (f) KEGG enrichment statistics of IG-3d vs. CK-3d in non-overlapping DEGs of IG-3h vs. CK-3h and IG-3d vs. CK-3d. (g) KEGG enrichment statistics of PG-3d vs. CK-3d in non-overlapping DEGs of PG-3d vs. CK-3d and IG-3d vs. CK-3d. (h) KEGG enrichment statistics of IG-3d vs. CK-3d in non-overlapping DEGs of PG-3d vs. CK-3d and IG-3d vs. CK-3d. Figure S2. GO analysis of DEGs during NO-dependent concentration regulation of embryo germination of *Sorbus pohuashanensis.* Figure S3. KEGG enrichment analysis of metabonomics in NO-dependent concentration regulation of *Sorbus pohuashanensis* embryo process.
