# Supplementary material for: Integrating Physiology, Transcriptome, and Metabolomics Reveals the Potential Mechanism of Nitric Oxide Concentration-Dependent Regulation of Embryo Germination in Sorbus pohuashanensis
Source: Plants (Basel). 2025 Jan 23;14(3):344. doi: 10.3390/plants14030344 (PMC11820237; doi:10.3390/plants14030344)
Supplement: Supplementary file 1 [file plants-14-00344-s001.zip › plants-3364213-supplementary.pdf]

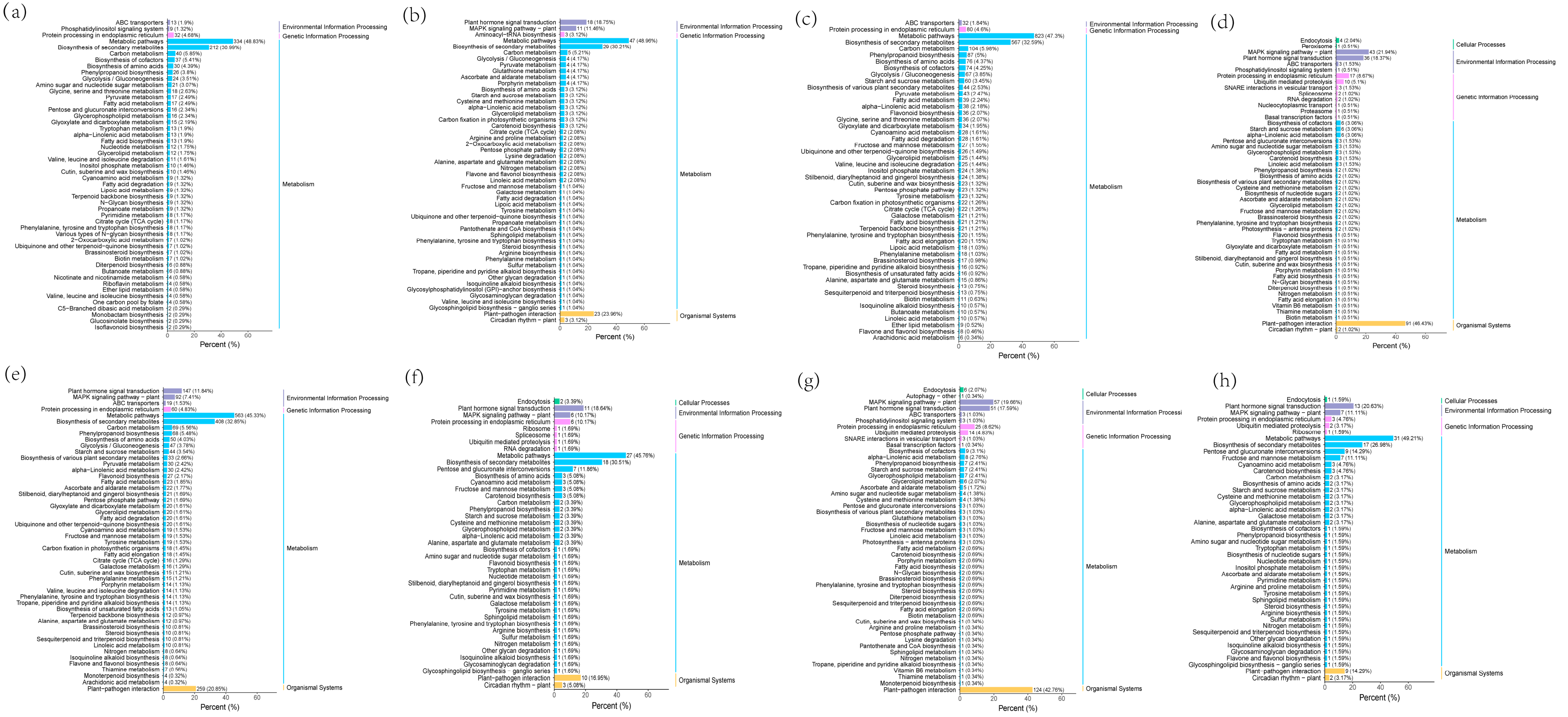

Figure S1. KEGG analysis of DEGs during NO-dependent concentration regulation of embryo germination of *Sorbus pohuashanensis*. (a) KEGG enrichment statistics of PG-3h vs CK-3h in non-overlapping DEGs of PG-3h vs CK-3h and IG-3h vs CK-3h. (b) KEGG enrichment statistics of IG-3h vs CK-3h in non-overlapping DEGs of PG-3h vs CK-3h and IG-3h vs CK-3h. (c) KEGG enrichment statistics of PG-3h vs CK-3h in non-overlapping DEGs of PG-3h vs CK-3h and PG-3d vs CK-3d. (d) KEGG enrichment statistics of PG-3d vs CK-3d in non-overlapping DEGs of PG-3h vs CK-3h and PG-3d vs CK-3d. (e) KEGG enrichment statistics of IG-3h vs CK-3h in IG-3h vs CK-3h and IG-3d vs CK-3d non-overlapping DEGs. (f) KEGG enrichment statistics of IG-3d vs CK-3d in non-overlapping DEGs of IG-3h vs CK-3h and IG-3d vs CK-3d. (g) KEGG enrichment statistics of PG-3d vs CK-3d in non-overlapping DEGs of PG-3d vs CK-3d and IG-3d vs CK-3d. (h) KEGG enrichment statistics of IG-3d vs CK-3d in non-overlapping DEGs of PG-3d vs CK-3d and IG-3d vs CK-3d.

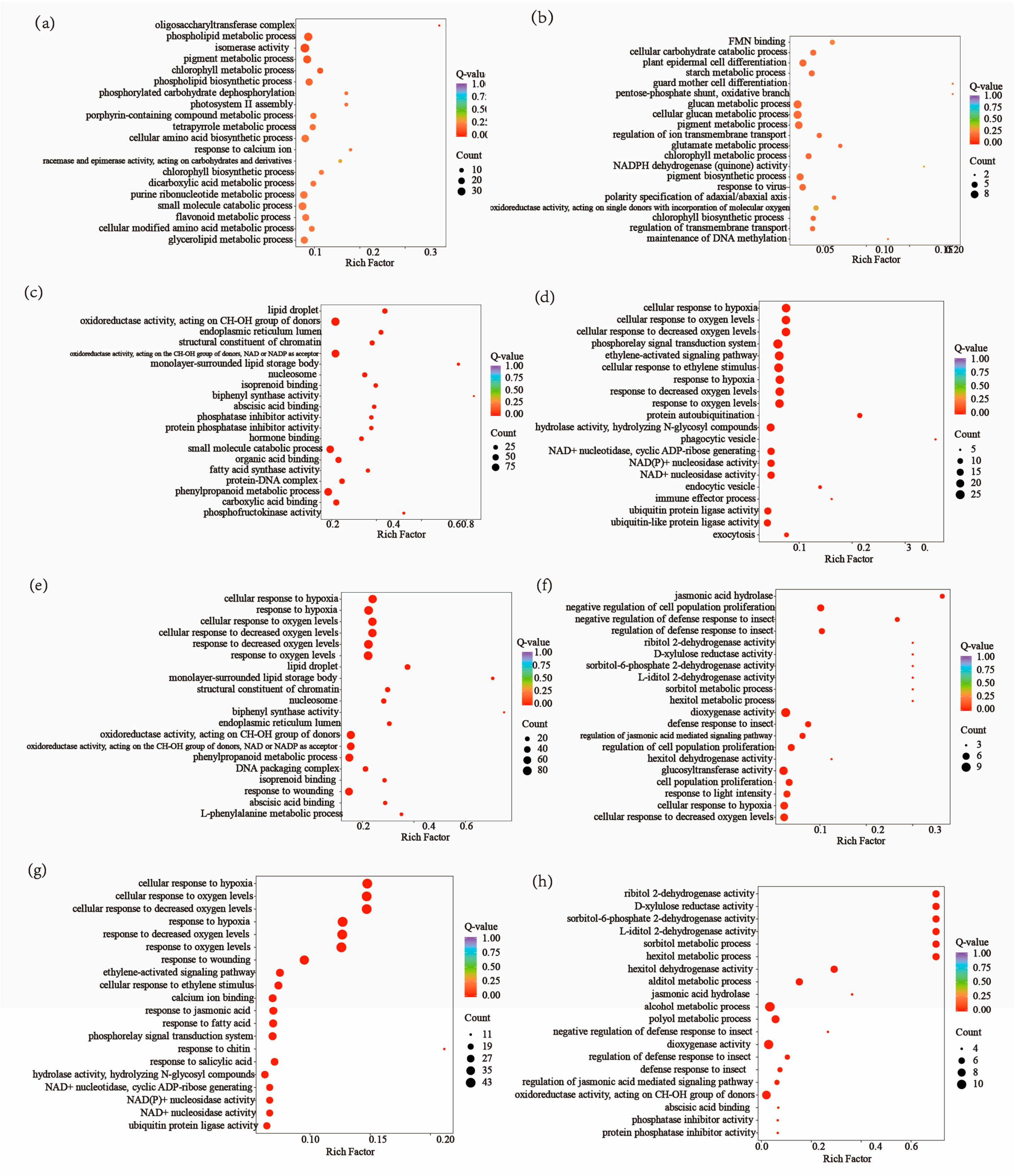

Figure S2. GO analysis of DEGs during NO-dependent concentration regulation of embryo germination of *Sorbus pohuashanensis*.

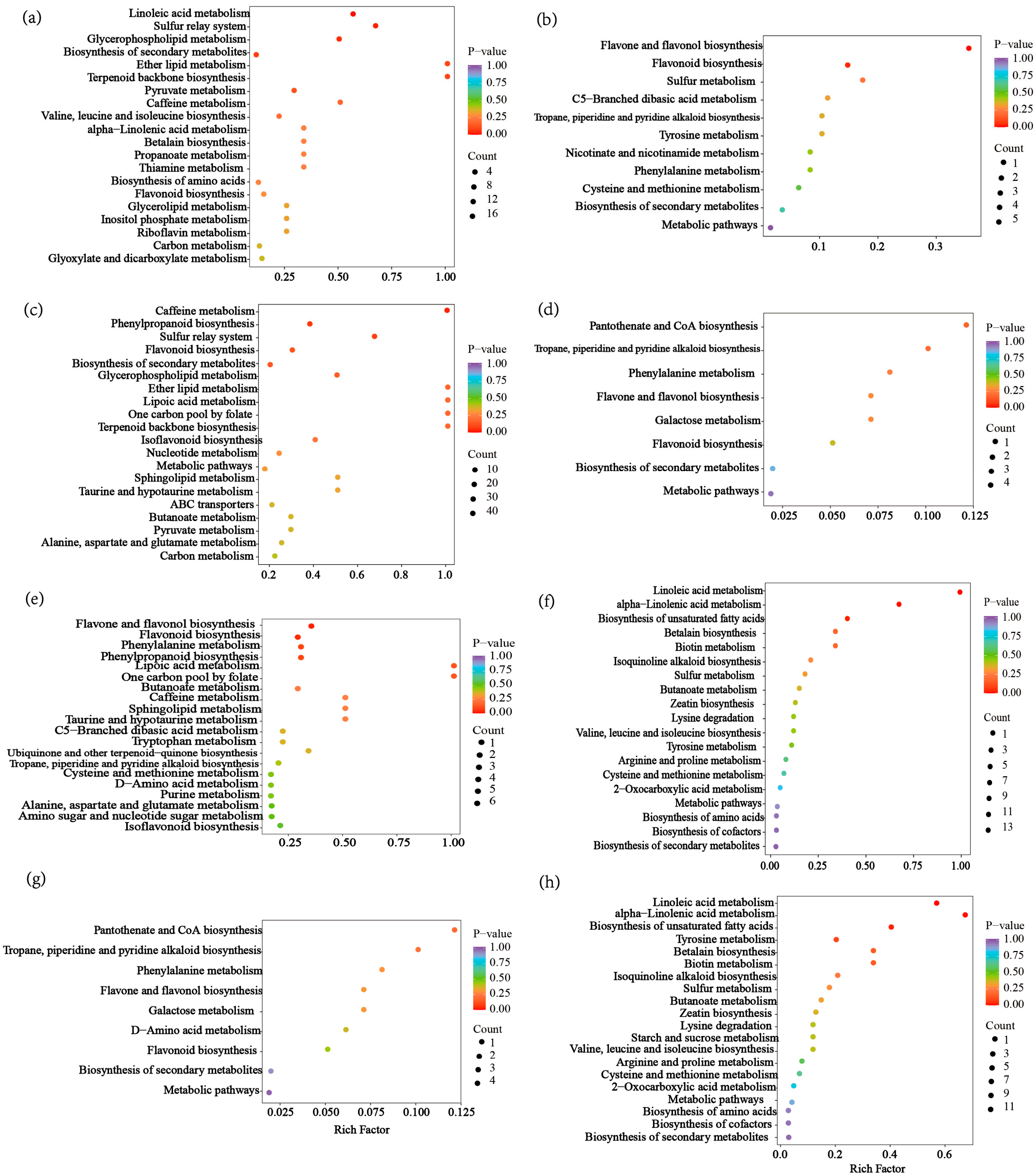

Figure S3. KEGG enrichment analysis of metabonomics in NO-dependent concentration regulation of *Sorbus pohuashanensis* embryo process.
